# The impact of neighborhood deprivation on patients’ unscheduled out-of-hours healthcare seeking behavior: a cross-sectional study

**DOI:** 10.1186/1471-2296-14-136

**Published:** 2013-09-14

**Authors:** Sara Willems, Wim Peersman, Philippe De Maeyer, Walter Buylaert, Jan De Maeseneer, Peter De Paepe

**Affiliations:** 1 Department of Family Medicine and Primary Health Care, Ghent University, 9000 Ghent, Belgium; 2 Department of Geography, Ghent University, 9000 Ghent, Belgium; 3 Department of Internal Medicine, Ghent University, 9000 Ghent, Belgium; 4 Heymans Institute of Pharmacology, Ghent University, 9000 Ghent, Belgium

**Keywords:** Out of hours care, Health care delivery, Emergency care, Primary health care, Primary care centre, Social class, Neighborhood deprivation

## Abstract

**Background:**

The use of unscheduled out of hours medical care is related to the social status of the patient. However, the social variance in the patient’s preference for a hospital based versus a primary care based facility, and the impact of specific patient characteristics such as the travel distance to both types of facilities is unclear. This study aims to determine the social gradient in emergency care seeking behavior (consulting the emergency department (ED) in a hospital or the community-based Primary Care Center (PCC)) taking into account patient characteristics including the geographical distance from the patient’s home to both services.

**Methods:**

A cross-sectional study, including 7,723 patients seeking out-of-hours care during 16 weekends and 2 public holidays was set up in all EDs and PCCs in Ghent, Belgium. Information on the consulted type of service, and neighborhood deprivation level was collected, but also the exact geographical distance from the patient’s home to both types of services, and if the patient has a regular GP.

**Results:**

Patients living in a socially deprived area have a higher propensity to choose a hospital-based ED than their counterparts living in more affluent neighborhoods. This social difference persists when taking into account distance to both services, having a regular GP, and being hospitalized or not. The impact of the distance between the patient’s home address and the location of both types of services on the patient’s choice of service is rather small.

**Conclusions:**

Initiatives aiming to lead patients more to PCC by penalizing inappropriate ED use might increase health inequity when they are not twinned with interventions improving the access to primary care services and tackling the underlying mechanisms of patients’ emergency care seeking behavior. Further research exploring the impact of out-of-hours care organization (gatekeeping, payment systems, …) and the patient’s perspectives on out-of-hours care services is needed.

## Background

International literature shows that low-income patients, patients with a low educational attainment, and the unemployed more frequently seek out-of-hours emergency care than do their more affluent counterparts [1-3]. This can partially be explained by a social gradient in the prevalence of disease (i.e. a step-wise increasing prevalence of disease when descending the social ladder). Patients with a low socioeconomic status (SES) also more often develop complications and reach the more severe stages of disease [4]. In some studies, the direction of the negative relationship between the SES and the frequency of emergency care utilization even becomes positive when morbidity is taken into consideration in multivariate models [5]. However healthcare organizations should also be taken into account when trying to explain these higher utilization rates; for example, barriers in the accessibility of regular care, postponed payment in out-of-hours care versus direct payment during the daytime [6].

When low-SES patients seek emergency care, most studies indicate that they prefer the hospital Emergency Department (ED) as a point of service compared with Primary Care (PC) services [7-10]. However, a limited number of studies report a higher preference for PC services [11]. These different findings are considered as reflecting differences in local context variables. In this regard, two examples from the United States are worth being considered. Cunningham et al. found that longer waiting times and a lower proportion of home visits in out-patient services increase the probability of poor people choosing the ED more than they increase the probability of higher-income patients choosing the ED [12]. In a study conducted in Dallas, the development of a healthcare network for the underserved population resulted in a greater preference for PC in the population of low-income families [11].

A patient’s choice between the ED and out-of-hours PC services might also be determined by the geographical distance to the facilities [13]. However, only a limited number of studies take distance to emergency care services into consideration when studying patients’ healthcare seeking behavior [3,13-15]. Furthermore, most of these studies have some important limitations, as they neither analyze healthcare seeking behavior at the individual level but at an aggregated level (e.g. the yearly consultation rates of an elective area), nor are they able to calculate the exact geographical distance from the home address of the patient to the different types of services. In addition, many studies limit their analysis to the utilization rates of only one type of service (e.g. ED) without taking into consideration the availability of other types of services (e.g. PC services).

The aim of the present study is to describe the use of unscheduled, direct out of hours care (at the Emergency Department in a hospital or the community-based Primary Care Centre) in the city of Ghent and to determine the social gradient in the use of this type of health care, taking into account patient characteristics, having a regular GP, and the exact geographical distance from the patient’s home to both services.

## Methods

### Design and setting

This observational, cross-sectional study was carried out in Ghent, the third-largest city of Belgium. Ghent covers 158 km^2^ with a population of approximately 247,000 residents.

During the weekend and holidays medical care is provided by primary care centers (PCC) and by emergency departments (ED) in hospitals. The PCCs are not attached to hospitals and are positioned centrally in the city. They are open during the weekend from 7 PM on Friday until 7 AM on Monday. During public holidays, the PCCs open the day before the holiday at 7 PM and close on the holiday after 7 AM. During opening hours, 3 or 4 GPs provide primary care for half the population of Ghent. GPs are on duty during 7 or 8 weekends per year for a 12-hour shift. When patients wish to consult a GP during out of hours, they dial a central telephone number. The secretary is in charge of the triage: mobile patients are asked to come to the nearest PCC. Immobile patients who are too ill to come to the PCC can be seen by a doctor at home. In the Belgian fee-for-service system, patients have to pay the GP immediately after the consultation. Although a major part of this expense is reimbursed by the sickness fund, patients have to advance the full amount at the moment of consultation (precise amounts presented in Table 1).

**Table 1:** Examples of the total cost for the patient, the reimbursement and the amount not reimbursed when consulting a GP or an ED during the weekend or on public holidays (amounts in euros)

	**Cost of the consultation**	**Amount reimbursed by sickness fund**	**Amount not reimbursed by sickness fund**
**GP**			
In the PCC between 8 AM and 9 PM	36.23	30.23	6
In the PCC between 9 PM and 8 AM	48.30	42.30	6
Home visit between 8 AM and 9 PM	54.39	35.66	18.73
Home visit between 9 PM and 8 AM	83	54.39	28.61
**Emergency department hospital**			
Without a referral letter from a GP	45.44 *	25.51	19.93
With a referral letter from a GP	45.44 *	41	4.44

*these amounts easily rise because of a higher likelihood for additional tests, technical interventions, or consultation with other specialists.

In the city of Ghent, four hospitals have a certified ED: one university hospital, two private hospitals, and one public hospital. All of them are directly accessible to all patients. The fee for consulting an emergency physician in the ED at night or during weekends is 45.44 euro but the cost easily rises because of a higher likelihood for additional tests, technical interventions, or consultation with other specialists. In the ED patients do not pay immediately: the hospital bills the patient the cost share part some time after the consultation (one week to months later). The reimbursable part of the cost is directly billed by the hospital to the patient’s sickness fund.

### Inclusion of participants

The study population were all patients seeking out of hours care without a referral in the EDs or the PCCs in the city of Ghent within a time frame of 12 months. Because of limited resources, data were collected during 16 randomly selected weekends, stratified by season, and 2 public holidays (one in winter, one in summer).

Because this study focuses on which service patients choose when they are in charge of this decision, patients referred to the ED by their GP were excluded. Also patients admitted to the ED via the emergency medical services (the European 112 number) by an ambulance (e.g. after a car accident) and patients transferred from another hospital or brought in by the police services were excluded from the study as for the majority of these patients someone else took the decision to go to the ED. In addition, patients undergoing chemotherapy or transplant patients were excluded, as they are usually instructed by their treating specialist to present to the ED in case of complications during out of hours. Also patients without a legal status in Belgium were excluded. Since they are not covered by the regular health insurance, access to the ED is administratively easier than going to the PCCs resulting in much higher utilization rates. Patients receiving home visits by the GP were also excluded because the impact of distance on patient’s emergency care seeking behavior could not be studied for these patients. Patients living outside the city of Ghent were excluded since information on the use of GP services located outside the city of Ghent was not available. Finally, patients were included in the database only once; if they consulted more than once in the reference period, then only the information of the first visit was included. Firstly, this was done to create a sample without outliers of frequently consulting patients weighing heavily on the analysis. Secondly, the choice of health care provider a second time can be influenced by an earlier contact (e.g. the GP telling the patient to consult the ED directly in a next event of need for health care). Thirdly, with this exclusion rule patients visiting the ED after a referral by the GP were excluded as their visit to the ED cannot be considered as a personal decision but is steered by their GP.

This resulted in an inclusion of 7,723 patients in the study.

### Data collection

All data needed for this study was collected retrospectively from the medical charts of the patients who consulted the EDs and the PCCs in the selected weekends/holidays and who fulfilled the inclusion criteria. Nine students from the Master in Medicine and the Master in Nursing program at Ghent University took up the function of chart abstractors. In order to improve the accuracy and to minimize inconsistencies in the chart reviews, a set of strategies was applied [16]. All chart abstractors were trained before the start of the data collection, using a set of “practice” medical records. They were instructed on how to select cases, how to define and code the variables and what to do in case of haziness. Standardized, electronic abstraction forms were used to guide data collection in order to ensure uniform handling of data that is conflicting, ambiguous, missing or unknown. Periodic meetings with chart abstractors and one of the promoters of the study were organized in order to resolve disputes and to review coding rules. The chart reviewers were not blind to the hypothesis being tested in this study. Before analyses names and exact birth dates were deleted for privacy reasons of the patients.

### Variables

Data on demographics, having a regular GP, home address, and time of consulting were collected from the patient charts. Distance and level of deprivation of the neighborhood in which the patient lives were calculated afterward based on the patient’s home address.

For the analysis, the following variables were composed:

Demographics: sex (male/female) and age. Age was categorized in 10 categories (in years): <1, 1–4, 5–14, 15–24, 25–34, 35–44, 45–54, 55–64, 65–74, 75 + .

When presenting in the ED and the PCC, patients are asked whether they have a regular GP and if so, the GP’s name is included in the chart. Having a GP is coded as “yes” when the name of the GP could be found in the patient’s medical chart.

The time of consulting in the ED or the PCC was categorized during either the night (7 PM – 7 AM) or the daytime (7.01 AM – 6.59 PM).

“Being hospitalized” indicates whether the patient is hospitalized as a result of his/her consultation in the ED (yes/no).

The distance in kilometers (kms) from the patient’s home address to the nearest PCC and the nearest ED was calculated. The home address is checked when patients arrive in the PCC or ED. The addresses of the four EDs and the two PCCs, and the patient’s home address were geocoded using geographical information system (GIS) software. Geocoding is a process in which data elements are imported and assigned geographic coordinates (latitude and longitude) that are used to calculate distances. The shortest distance in kms by road for a motorized vehicle and the shortest distance for a pedestrian were calculated, as were also both the shortest distances in minutes. In order to increase the relevancy of the outcomes of the analysis, three categories were formed: the ED is at least 25% closer to the patient’s home than the PCC; the ED and PCC are at the same distance (i.e. the ED is no more than 25% closer or 25% further than the PCC); and the ED is at least 25% further from the patient’s home than the PCC. To decide on the cut-off points of 25% both the relevancy and the sample sizes in the three categories were considered. As no differences were found in the results when using the distances for a pedestrian or for a motorized vehicle, only the latter were reported and used in the multivariate analysis.

The level of deprivation of the statistical sector in which the patient lives was used to determine the patient’s social status. The city of Ghent is divided into 201 statistical sectors of which 142 are populated (the other sectors are recreation areas, harbor, …). A statistical sector, which is comparable to the census tract level in the Anglo-Saxon system, is the smallest administrative level at which objective administrative data (demographic, social, and economic indicators) are available. We first determined the sector in which the patient lives, based on the patient’s home address. Next, the level of deprivation of the patients’ sector was defined according to the Atlas of Deprived Neighborhoods [17] and categorized into 4 categories: no deprivation, low level of deprivation, moderate level of deprivation, and high level of deprivation.

The Atlas of Deprived Neighborhoods contains for each statistical sector in Flanders and Brussels information on 22 variables originating from tax and census databases and describes the population composition (proportion of migrants, house-owners, single-parent families, educational level of the population, and number of unemployed and manual workers, …); the houses in the statistical sector (number of rooms in the house, quality of the house, and having central heating); and the quality of the physical environment (air pollution, noise, and garbage in the streets). Based on these variables seven indicators are built using principle component analysis. For each indicator a threshold defined by experts in the field is determined. Sectors which score under the threshold for at least 4 indicators, are labeled as deprived (i.e. 38 of the 142 sectors in Ghent). To determine the level of deprivation, a new principal component analysis is used to first cluster the deprived sectors in types of deprivation (11 types identified). Then, these types are ranked according to the number of indicators for which they score below the mean score of all types, and the extent to which they score under the mean score. According to this ranking, 3 types are defined as extremely deprived (high level of deprivation), 3 types as moderately deprived (moderate level of deprivation), and 3 as slightly deprived (low level of deprivation). Two types are not ranked since they are considered “a-typical” [17]. All deprived sectors in Ghent could be classified into one of the first nine types and therefore be reclassified as extremely deprived, moderately deprived, or slightly deprived. No neighborhoods were classified into one of the two “a-typical” types of neighborhoods.

The outcome variable in this study was the type of service chosen by the patient, that is, an ED or a PCC.

Data on legal status, consulting for complications of chemotherapy and admission by the ambulance and police were also collected from the charts and used to exclude patients from the study in case they were not excluded already upon entry in the ED.

### Ethics

This study was approved by the ethics committee of Ghent University Hospital (study registration number B67020072708).

### Analysis

First, to describe the study, sample frequencies were calculated using SPSS Statistics 19. Next, the relation between the patient’s choice of service and patient characteristics was studied by comparing percentages and using chi-square tests. Finally, to study the association between the patient’s choice and the deprivation level of the neighborhood, a logistic regression model was built with age, sex, having a regular GP, time of consulting, subsequent hospitalization, and distance included as potential confounders.

## Results

Table 2 describes the study population and the bivariate relationship between the type of service the patient visits when in need of out-of-hours care and patient characteristics.

**Table 2:** Characteristics of patients seeking out-of-hours care in the ED or in a PCC

	**Total sample**	**Patients attending the ED**	**Patients attending the PCC**	
	**n (% within total sample)**	**n (% of total sample)**	**n (% of total sample)**	**p-value**
Type of service used by the patient				
ED	3727 (48.3)			
PCC	3994 (51.7)			
Sex				<0.001
Male	3754 (48.6)	2012 (46.4)	1741 (53.6)	
Female	3969 (51.4)	1715 (43.2)	2253 (56.8)	
Missing	0			
Age group (years)				<0.001
<1	362 (4.7)	186 (51.4)	176 (48.6)	
1–4	902 (11.8)	402 (44.6)	500 (55.4)	
5–14	699 (9.1)	402 (57.5)	297 (42.5)	
15–24	1061 (13.8)	642 (60.5)	419 (39.5)	
25–34	1446 (18.9)	773 (53.5)	671 (46.5)	
35–44	964 (12.6)	506 (52.5)	458 (47.5)	
45–54	665 (8.7)	342 (51.4)	323 (48.6)	
55–64	447 (5.8)	201 (45.0)	246 (55.0)	
65–74	396 (5.2)	144 (36.4)	252 (63.6)	
75+	727 (9.5)	129 (17.7)	598 (82.3)	
Missing	54			
Having a regular GP				<0.001
Yes	6522 (84.5)	2845 (43.6)	3675 (56.4)	
No	1198 (15.5)	879 (73.4)	319 (26.6)	
Missing	3			
Being hospitalized				<0.001
Yes	619 (8.0)	578 (93.5)	40 (6.5)	
No	7104 (92)	3149 (44.3)	3954 (55.7)	
Missing	0			
Moment of consulting				<0.001
Day	4807 (62.3)	2107 (43.8)	2700 (56.2)	
Night	2914 (37.7)	1618 (55.6)	1294 (44.4)	
Missing	2			
Distance				<0.001
ED at least 25% closer than PCC	2343 (30.3)	1285 (54.8)	1058 (45.2)	
ED and PCC at the same distance	3803 (49.2)	1740 (45.8)	2063 (54.2)	
ED at least 25% further than PCC	1577 (20.4)	702 (44.6)	873 (51.7)	
Level of deprivation of the neighborhood where the patient lives				<0.001
Not deprived	5034 (65.2)	2183 (43.4)	2850 (56.6)	
Low level of deprivation	1482 (19.2)	822 (55.5)	660 (44.5)	
Moderate level of deprivation	988 (12.8)	561 (56.8)	426 (43.2)	
High level of deprivation	219 (2.8)	161 (73.5)	58 (26.5)	
Missing	0			
Total	7723 (100)	3994 (51.7)	3727 (48.3)	

ED: Emergency Department in the hospital; PCC: community-based Primary Care Center; GP: general practitioner.

### Characteristics of study subjects

Of all the patients seeking out-of-hour care during the study period, 48.3% went to an ED (n = 3727), and 51.7% (n = 3994) went to a PCC. The majority of the patients sought care during the day (62.3%, n = 4807) and were not hospitalized after the consultation (92.0%, n = 7104).

Slightly more patients were women (51.4%; n = 3969). When compared to the other age groups a higher proportion of children between 1 and 4 years old, adolescents and young adults between 15 and 24, adults between 25 and 35, and adults between 35 and 45 (respectively 11.8%, 13.8%, 18.9%, and 12.6%) was found in the sample. A vast majority of the patients reported having a regular GP (84.5%, n = 6522). Nearly 35% (34.8%, n = 2689) of the consulting patients live in one of the deprived neighborhoods of Ghent: 219 (2.8%) in a heavily deprived neighborhood, 988 (12.8%) in a moderately deprived neighborhood, and 1482 (19.2%) in a slightly deprived neighborhood.

About half of the patients live nearly as close to an ED as to a PCC (49.2%, n = 3803), 30.3% (n = 2343) live closer to an ED than to a PCC, and 20.4% (n = 1577) live closer to a PCC than to an ED, when using the travel time in kms by motorized vehicle as the measure. The average distance from the patients’ home to the nearest PCC is 3.29 kms (SD ± 1,84) and to the nearest ED is 2.91 kms (SD ± 1,83).

### The relation between the service the patient visits and patient characteristics

When in need for out-of-hours medical care, 43.2% of the women and 46.4% of the men go to the ED (p < 0,001). Age is also significantly related to going to the emergency care (p < 0.001). Especially in the two oldest patient groups patients go less to an ED: 36.7% of the 65–74-year olds and 17.7% of the 75+ year olds go to the ED. Having a regular GP is significantly associated with going to a PCC (p < 0,001). Almost three quarters (73.4%) of the patients without a regular GP prefer an ED, which is in contrast with 43.6% of the patients with a regular GP.

Patients living closer to an ED than to a PCC (measured in kms by motorized vehicle) go more often to an ED than to a PCC (54.8% versus 45.2%). Patients living as close to an ED as to a PCC and patients living closer to a PCC go more often to a PCC (respectively 45.8% versus 54.2%, and 44.6% versus 51.7%). The higher the level of deprivation of the neighborhood in which the patient lives, the more patients go to an ED compared with a PCC (p < 0,001: 43.4% of the patients living in a not deprived neighborhood; 55.5% of the patients living in a neighborhood with a low level of deprivation; 56.8% of the patients living in a neighborhood with a moderate level of deprivation; and 73.5% of the patients living in a neighborhood with a high level of deprivation go to an ED when they are in need of out-of-hours care. Finally, during daytime hours, the majority of the patients go to a PCC (56.2%); whereas at nighttime, the majority go to an ED (55.6%) (p < 0.001).

### Multivariate analysis of the impact of neighborhood deprivation

To determine the independent impact of the deprivation of the patient’s neighborhood on the type of service consulted during out of hours, a logistic regression model was built by adjusting for the patient characteristics included in Table 3. It demonstrates that the deprivation level of the neighborhood in which the patient lives has an impact on the type of service the patient chooses, even when adjusting for sex, age group, having a regular GP, being hospitalized (as an outcome of the consultation), the time of consultation, and the distance between the patient’s home address and both services.

**Table 3:** The contribution of the neighborhood deprivation level on the out-of-hours healthcare seeking behavior (going to the ED or to the PCC): logistic regression model

	**Odds ratio**	**95% CI**	**P-value**
Sex			
Female	1		
Male	1.37	1.24 – 1.51	<0.001
Age group (years)			
0–1	5.65	4.03 – 7.94	<0.001
1–4	5.44	4.09 – 7.25	<0.001
5–14	10.43	7.76 – 14.00	<0.001
15–24	10.85	8.19 – 14.37	<0.001
25–34	8.08	6.15 – 10.61	<0.001
35–44	8.12	6.12 – 10.78	<0.001
45–54	7.40	5.49 – 9.96	<0.001
55–64	6.02	4.36 – 8.30	<0.001
65–74	2.99	2.12 – 4.23	<0.001
75+	1		
Having a regular GP			
Yes	1		
No	3.25	2.80 – 3.78	<0.001
Being hospitalized			
No	1		
Yes	34.16	23.89 – 48.83	<0.001
Time of consulting			
Day	1		
Night	1.52	1.37 – 1.69	<0.001
Distance			
ED at least 25% closer than PCC	1.30	1.12 – 1.50	0.001
ED and PCC at the same distance	1.03	0.90 – 1.18	0.618
ED at least 25% further than PCC	1		
Level of deprivation of the neighborhood where the patient lives			
Not deprived	1		
Low level of deprivation	1.60	1.40 – 1.83	<0.001
Moderate level of deprivation	1.47	1.26 – 1.71	<0.001
High level of deprivation	2.57	1.84 – 3.58	<0.001

ED: Emergency Department in the hospital; PCC: community-based Primary Care Center; GP: general practitioner.

The odds ratio for consulting an ED when living in a neighborhood with a high level of deprivation is 2.57, which represents a higher use of the ED for patients living in these neighborhoods compared with patients living in a not deprived neighborhood (95% CI: 1.84 – 3.58). The odds ratio for consulting an ED when living in a neighborhood with a moderate level of deprivation is 1.47 (95% CI: 1.26 – 1.71) and when living in a neighborhood with a low level of deprivation is 1.60 (95% CI: 1.40 – 1.83) compared with patients living in a not deprived neighborhood.

Analysis using the time of traveling to the services by foot instead of by motorized vehicle did not result in different findings (details not reported).

## Discussion

Research shows that the choice of type of unscheduled out-of-hours health care (hospital-based versus primary care setting) is socially determined [7-11]. However, the strength and even the direction of the relation seems to be highly dependent on other patient characteristics [11-13]. In this context, the availability of services and distance between the patient’s home and both types of services is suggested as a confounding factor [13]. However, most published studies do not provide information on distance or are limited because they use information at population level and not on patient level.

Our study exhibits a clear social difference in unscheduled out-of-hours healthcare seeking behavior even when taking into account differences in the distance from the patient’s home to both types of services, in having a regular GP, and in being hospitalized or not. Patients living in a socially deprived area have a higher propensity to choose a hospital-based ED when being confronted with medical problems out of hours than their counterparts living in more affluent neighborhoods. This difference is not only most pronounced for those living in strongly deprived areas but also holds for patients living in neighborhoods with a moderate level or a low level of deprivation.

A UK based study reported that the impact of distance between the patient’s home and out of hours services completely disappears when taking deprivation into account.^3^ In the present study, where distances are measured in great detail and on patient level, its impact on the patient’s health care seeking behavior does not completely disappear. Although the geographical accessibility is of great importance in achieving equitable health care, its relevance probably diminishes in a context where citizens live in near proximity of both services (in this study on average 3.3 kms from a PCC and 2.9 kms from an ED, in a city with good public transport and a high walkability index) [18]. Other service characteristics might have influenced the accessibility of both services more importantly resulting in a higher use of EDs for people living in deprived neighborhoods.

Hospital EDs become more attractive sources of non-urgent care because of their convenience [18]. In Belgium this more specifically might be true regarding the convenience of the payment system. In the Belgian fee-for-service system, where patients have to pay a GP in the PCC immediately after the consultation but receive the hospital bill for ED care some time after the consultation, the urgent problem of needing medical care and having no money is solved when consulting the ED. Out-of-hours care might therefore, appeal more to deprived patients whose living conditions force them to adopt a short-term perspective that is focused on survival and rapid problem-solving. A quantitative, prospective study could test this hypothesis.

Access to care is not only a matter of barriers in the organization of care but also a matter of patients’ knowledge and perceptions about the organization and the quality of care. Differences in these aspects between social groups might result in social differences in healthcare seeking behavior. For example, patient’s preference for the ED is reported to be associated with higher levels of trust in the delivered care at EDs [19], which is characterized by more tests and technological interference. In addition, patients from deprived patient groups have lower levels of health literacy and healthcare literacy [20]. In several European countries, the sector of out-of-hours primary care has experienced important changes in the past decades [21,22]. In the city of Ghent, a system of locally organized care by groups of collaborating doctors delivering most out-of-hours care during home visits has evolved into a central organized system with a central telephone number and care mainly delivered in a primary care center where patients come in to get medical care. The penetration of knowledge about this relatively new healthcare system might be different for patients from different social classes, reaching the better-off and well-educated most. To meet these needs, the city of Ghent recently set up a major campaign that promotes the use of the PCC. Information on the PCCs was also distributed through community centers, social services, and local organizations in order to also reach the most deprived and isolated inhabitants of Ghent. It would be interesting to repeat this study after this campaign, including a measurement of the knowledge of patients about the PCCs and EDs. Also more qualitative studies exploring patients’ opinions and experiences could lead to more insights. They are needed to further identify factors that direct lower SES patients to hospital-based out of hours care.

This study confirms the findings of other studies that describe the positive relationship between having a regular GP and the use of ED out-of-hours health care [23-25]: the odds ratio for patients without a regular GP to go to an ED is 3.25 compared to patients with a regular GP even after controlling for other patient characteristics such as social status of the patient. This seems to indicate that increasing the number of patients with a regular GP could have effects on healthcare utilization patterns of the population.

An important limitation of this study is that the severity of the patient’s condition is not included as a covariate in the analysis of his/her health care seeking behavior. One could expect that the lower SES patients’ higher propensity to go to an ED is partly due to higher medical urgency. It is known that patients from lower SES are later diagnosed with severe conditions resulting in a higher prevalence of the more advanced stages of disease [26-28], and present with more multimorbidity than patients from higher SES [29]. In this study the ICPC (International Classification of Primary Care) code for the reason for encounter and the diagnosis was available. However for most ICPC codes, the level of detail is not sufficient to serve as an indicator of severity (e.g. A92 – Allergic reaction). For the limited number of codes that potentially could be used as a proxy for severity (e.g. K75 – Acute myocardial infarction) the frequencies were too low for subanalysis. Hospitalization was also included in the database. However the relation between being hospitalized and the severity of the patient’s condition is unclear and might be more determined by the individual physician's characteristics [30].

An important merit of this study is the fact that all patients seeking care in one of the four EDs or in one of the two PCCs during the research period in the city of Ghent and who met the inclusion criteria were included in the study. This resulted in a relatively large study sample in a clearly defined geographical region. Nevertheless, we did not include all patients seeking unscheduled out-of-hours care in Ghent. One smaller hospital in a suburb of Ghent was not included in this study. This hospital does not have a certificated ED but provides some first aid care; however, this is limited to about three to five patients per weekend day. Although all GPs in Ghent participate in the PCCs out-of-hours care organization, it is possible that some GPs provided some emergency care outside the PCCs.

Another important strength is that for all the included patients, the exact distances to the services could be calculated. However, although the calculation of the distances itself was performed very accurately, the home address was used as a base that might prove to be a bias. Patients do not always travel from home when they seek unscheduled out of hours care. It is possible that the patient does not live at his/her official home address or got hurt at another place. This is especially the case for trauma and injuries and might be the explanation of the relatively higher ED use of people between 15 and 35 years.

To determine the socioeconomic status of the patient, area level scores of deprivation are used as a proxy. Caution should be paid in the interpretation of the results, especially when assuming that the associations observed at the area level reflect the same association at the individual level. This may not be true, a problem known as ecological fallacy [31].

## Conclusions

Healthcare systems are under increasing pressure: they are challenged to work more cost effectively in a context where the demands on the system increase. In debates on this topic tackling the inappropriate use of EDs is a recurrent item [32]. Leading patients to the most appropriate point of service might indeed lower the pressure on out-of-hours medical care. However, studies like ours, which report a strong social gradient in healthcare use, might be used as an argument to mainly focus on the lower-educated and most vulnerable patient groups. Measures aiming at changing out-of-hours care behavior should, indeed, recognize this social gradient in healthcare utilization. However, this cannot be done without taking into account that EDs may serve as a safety net for socially deprived patients who encounter problems in the accessibility of the primary healthcare system. In this context, initiatives such as the Washington State’s Health Care Authority intention to stop the payment of ED visits for vulnerable population groups “when those visits are not necessary for that place of service” [33] might increase the inequity in health unless they are twinned with interventions to improve the access to out-of-hours primary care services.

First, a prerequisite of patients consulting primary care when they need out-of-hours medical care is the availability of primary care services. In some countries, such as Belgium, the number of doctors in EDs significantly exceeds the number of available GPs.

Second, patients need an insight into the organization of the out-of-hours care and know where and how to turn to the primary care facilities. While informing patients about the existence of PCCs when they consult for primary care sensitive conditions in the ED might change their future utilization pattern.

Third, primary care services should not have financial barriers: cost-share should be reduced to a minimum, and systems in which patients advance the total fee with reimbursement some time later should be avoided.

Further research is needed to test the findings of our study. Although we did not find an impact of distance on the patient’s choice between the ED and the PCC, this is not necessarily true for countries or areas where the population density or service density is much lower and distances to care facilities are much larger. In addition, distances might become of more importance when barriers in the access to one of both types of services are different. Finally, the results of studies that describe a patient’s utilization patterns win interest when they can be compared with an exploration of a patient’s perspectives.

## Abbreviations

CI: Confidence interval; ED: Emergency department; GP: General practitioner; kms: Kilometers; PCC: Primary care centre.

## Competing interests

All authors declare not to have received support from any organization for the submitted work; nor to have any financial relationships with any organizations that might have an interest in the submitted work in the previous 3 years; nor to have other relationships or activities that could appear to have influenced the submitted work.

## Authors’ contributions

WB, PDP, JDM were involved in the conception of the study. All authors were involved in the design of the study, the analysis and interpretation of data. SW drafted the article, all other authors revised it critically for important intellectual content. All authors gave final approval of the version to be published.

## Authors’ information

The design and preliminary results of the study reported in this paper were presented at the Symposium of the Belgian Society of Emergency and Disaster Medicine (BESEDIM), Brussels, January 2010.

No grant was obtained for this study.

## Pre-publication history

The pre-publication history for this paper can be accessed here:

http://www.biomedcentral.com/1471-2296/14/136/prepub

## References

[B1] Drummond N McConnachie A O'Donnell CA Moffat KJ Wilson P Ross S Social variation in reasons for contacting general practice out-of-hours: implications for daytime service provision? Br J Gen Pract 2000 50 460 464 10962783 PMC1313723

[B2] Majeed FA Cook DG Hilton S Poloniecki J Hagen A Annual night visiting rates in 129 general practices in one family health services authority: association with patient and general practice characteristics Br J Gen Pract 1995 45 531 535 7492422 PMC1239404

[B3] Carlisle R Groom LM Avery AJ Boot D Earwicker S Relation of out of hours activity by general practice and accident and emergency services with deprivation in Nottingham: longitudinal survey BMJ 1998 316 520 523 10.1136/bmj.316.7130.520 9501715 PMC2665670

[B4] McWilliams JM Zaslavsky AM Meara E Ayanian JZ Health insurance coverage and mortality among the near-elderly Health Aff (Millwood) 2004 23 223 233 10.1377/hlthaff.23.4.223 15318584

[B5] Burström B Increasing inequalities in health care utilization across income groups in Sweden during the 1990s? Health Policy 2002 62 117 129 10.1016/S0168-8510(02)00016-7 12354407

[B6] Droomers M Westert GP Do lower socioeconomic groups use more health services, because they suffer from more illnesses? Eur J Public Health 2004 14 311 313 10.1093/eurpub/14.3.311 15369040

[B7] O'Brien GM Stein MD Zierler S Shapiro M O'Sullivan P Woolard R Use of the ED as a regular source of care: associated factors beyond lack of health insurance Ann Emerg Med 1997 30 286 291 10.1016/S0196-0644(97)70163-X 9287889

[B8] Gordon JA Chudnofsky CR Hayward RA Where health and welfare meet: social deprivation among patients in the emergency department J Urban Health 2001 78 104 111 10.1093/jurban/78.1.104 11368190 PMC3456193

[B9] Norredam M Krasnik A Moller Sorensen T Keiding N Joost Michaelsen J Sonne Nielsen A Emergency room utilization in Copenhagen: a comparison of immigrant groups and Danish-born residents Scand J Public Health 2004 32 53 59 10.1080/14034940310001659 14757549

[B10] Rué M Cabré X Soler-González J Bosch A Almirall M Serna MC Emergency hospital services utilization in Lleida (Spain): A cross-sectional study of immigrant and Spanish-born populations BMC Health Serv Res 2008 8 81 10.1186/1472-6963-8-81 18402704 PMC2329626

[B11] Dehaven M Kitzman-Ulrich H Gimpel N Culica D O'Neil L Marcee A Foster B Biggs M Walton J The effects of a community-based partnership, Project Access Dallas (PAD), on emergency department utilization and costs among the uninsured J Public Health 2012 34 577 583 10.1093/pubmed/fds027 22653885

[B12] Cunningham PJ What accounts for differences in the use of hospital emergency departments across U.S. communities? Health Aff (Millwood) 2006 25 324 336 10.1377/hlthaff.25.w324 16849363

[B13] Lee JE Sung JH Ward WB Fos PJ Lee WJ Kim JC Utilization of the emergency room: impact of geographic distance Geospat Health 2007 1 243 253 18686249 10.4081/gh.2007.272

[B14] Ingram DR Clarke DR Murdie RA Distance and the decision to visit an emergency department Soc Sci Med 1978 12 55 62 10.1016/0160-8002(78)90007-2 644349

[B15] Magnusson G The hospital emergency department as the primary source of medical care Scand J Soc Med 1980 8 149 156 7209458 10.1177/140349488000800311

[B16] Gilbert E Lowenstein S Koziol-McLain J Barta J Steiner J Chart reviews in emergency medicine research: where are the methods? Ann Emerg Med 1996 27 305 308 10.1016/S0196-0644(96)70264-0 8599488

[B17] Vandermotten C Marissal P Van Hamme G Kesteloot C Slegers K Vanden Broucke L Ippersiel B de Bethune S Naiken R Dynamische analyse van de buurten in moeilijkheden in de Belgische stadsgewesten 2006 Brussels: POD Maatschappelijke Integratie, Armoedebestrijding, Sociale Economie en Grootstedenbeleid

[B18] Guttman N Zimmerman DR Nelson MS The many faces of access: reasons for medically nonurgent emergency department visits J Health Polit Policy Law 2003 28 1089 1120 10.1215/03616878-28-6-1089 14756500

[B19] Sempere-Selva T Peiró S Sendra-Pina P Martínez-Espín C López-Aguilera I Inappropriate use of an accident and emergency department: magnitude, associated factors, and reasons-an approach with explicit criteria Ann Emerg Med 2001 37 568 579 10.1067/mem.2001.113464 11385325

[B20] Kutner M Greenberg E Jin Y Paulsen C The health literacy of American’s adults: results from the 2003 National Assessment of Adult Literacy (NCES 2006–483) 2006 Washington, DC: National Center for Education Statistics

[B21] Giesen P Smits M Huibers L Grol R Wensing M Quality of after-hours primary care in the Netherlands: a narrative review Ann Int Med 2011 155 108 113 10.7326/0003-4819-155-2-201107190-00006 21768584

[B22] van Uden CJT Crebolder HFJM Does setting up out of hours primary care cooperatives outside a hospital reduce demand for emergency care? Emerg Med J 2004 21 722 723 10.1136/emj.2004.016071 15496709 PMC1726502

[B23] Philips H Remmen R De Paepe P Buylaert W Van Royen P Out of hours care: a profile analysis of patients attending the emergency department and the general practitioner on call BMC Fam Pract 2010 11 88 10.1186/1471-2296-11-88 21078162 PMC2998456

[B24] van Charante EP M ter Riet G Bindels P Self-referrals to the A&E department during out-of-hours: Patients’ motives and characteristics Patient Educ Couns 2008 70 256 265 10.1016/j.pec.2007.10.012 18063340

[B25] Moll van Charante EP van Steenwijk-Opdam PCE Bindels PJE Out-of-hours demand for GP care and emergency services: patients’ choices and referrals by general practitioners and ambulance services BMC Fam Pract 2007 8 46 10.1186/1471-2296-8-46 17672915 PMC2082275

[B26] Rannestad T Skjeldestad FE Socioeconomic conditions and number of pain sites in women BMC Womens Health 2012 12 7 10.1186/1472-6874-12-7 22458415 PMC3350397

[B27] Rapiti E Fioretta G Schaffar R Neyroud-Caspar I Verkooijen HM Schmidlin F Miralbell R Zanetti R Bouchardy C Impact of socioeconomic status on prostate cancer diagnosis, treatment, and prognosis Cancer 2009 115 5556 5565 10.1002/cncr.24607 19787636

[B28] Neuburger J Hutchings A Allwood D Black N van der Meulen JH Sociodemographic differences in the severity and duration of disease amongst patients undergoing hip or knee replacement surgery J Public Health 2012 34 421 429 10.1093/pubmed/fdr119 22267293

[B29] Schafer I Hansen H Schön G Höfels S Altiner A Dahlhaus A Gensichen J Riedel-Heller S Weyerer S Blank WA König HH von dem Knesebeck O Wegscheider K Scherer M van den Bussche H Wiese B The influence of age, gender and socio-economic status on multimorbidity patterns in primary care. First results from the multicare cohort study BMC Health Serv Res 2012 12 89 10.1186/1472-6963-12-89 22471952 PMC3348059

[B30] Gaucher N Bailey B Gravel J Impact of physicians’ characteristics on the admission risk among children visiting a pediatric emergency department Pediatr Emerg Care 2012 28 120 124 22270494 10.1097/PEC.0b013e318243f8e0

[B31] Sedgwick P The ecological fallacy BMJ 2011 343 d4670 10.1136/bmj.d4670 26391012

[B32] Durand A Gentile S Devictor B Palazzolo S Vignally P Gerbeaux P Sambuc R ED: patients: how nonurgent are they? Systematic review of the emergency medicine literature Am J Emerg Med 2011 29 333 345 10.1016/j.ajem.2010.01.003 20825838

[B33] Kellermann AL Weinick RM Emergency departments, Medicaid costs, and access to primary care – Understanding the Link N Engl J Med 2012 366 2141 2143 10.1056/NEJMp1203247 22591255

